# Glutamine Supplementation Alleviates Vasculopathy and Corrects Metabolic Profile in an In Vivo Model of Endothelial Cell Dysfunction

**DOI:** 10.1371/journal.pone.0065458

**Published:** 2013-06-11

**Authors:** Francesco Addabbo, Qiuying Chen, Dhara P. Patel, May Rabadi, Brian Ratliff, Frank Zhang, Jean-Francois Jasmin, Michael Wolin, Michael Lisanti, Steven S. Gross, Michael S. Goligorsky

**Affiliations:** 1 Departments of Medicine, Pharmacology and Physiology, New York Medical College, Valhalla, New York, United States of America; 2 Department of Experimental Immunopathology, National Institute of Gastroenterology, IRCCS “S. De Bellis” Castellana Grotte, Italy; 3 Department of Pharmacology, Weill Cornell Medical College, New York, New York, United States of America; 4 Cancer Center, Thomas Jefferson University, Philadelphia, Pennsylvania, United States of America; 5 Breakthrough Breast Cancer Research Unit, University of Manchester, United Kingdom; University of Colorado Denver, United States of America

## Abstract

Endothelial Cell Dysfunction (ECD) is a recognized harbinger of a host of chronic cardiovascular diseases. Using a mouse model of ECD triggered by treatment with L-Nω-methylarginine (L-NMMA), we previously demonstrated that renal microvasculature displays a perturbed protein profile, including diminished expression of two key enzymes of the Krebs cycle associated with a Warburg-type suppression of mitochondrial metabolism. We hypothesized that supplementation with L-glutamine (GLN), that can enter the Krebs cycle downstream this enzymatic bottleneck, would normalize vascular function and alleviate mitochondrial dysfunction. To test this hypothesis, mice with chronic L-NMMA-induced ECD were co-treated with GLN at different concentrations for 2 months. Results confirmed that L-NMMA led to a defect in acetylcholine-induced relaxation of aortic rings that was dose-dependently prevented by GLN. In caveolin-1 transgenic mice characterized by eNOS inactivation, L-NMMA further impaired vasorelaxation which was partially rescued by GLN co-treatment. Pro-inflammatory profile induced by L-NMMA was blunted in mice co-treated with GLN. Using an LC/MS platform for metabolite profiling, we sought to identify metabolic perturbations associated with ECD and offset by GLN supplementation. 3453 plasma molecules could be detected with 100% frequency in mice from at least one treatment group. Among these, 37 were found to be differentially expressed in a 4-way comparison of control vs. LNMMA both with and without GLN. One of such molecules, hippuric acid, an “uremic toxin” was found to be elevated in our non-uremic mice receiving L-NMMA, but normalized by treatment with GLN. Ex vivo analysis of hippuric acid effects on vasomotion demonstrated that it significantly reduced acetylcholine-induced vasorelaxation of vascular rings. In conclusion, functional and metabolic profiling of animals with early ECD revealed macrovasculopathy and that supplementation GLN is capable of improving vascular function. Metabolomic analyses reveal elevation of hippuric acid, which may further exacerbate vasculopathy even before the development of uremia.

## Introduction

Endothelial cell dysfunction (ECD) is a harbinger of multiple cardiovascular diseases, as diverse as atherosclerosis, hypertension, diabetes, to mention a few [1]. ECD is highly prevalent in chronic kidneys diseases (CKD) where it is responsible for a sharp increase in cardiovascular morbidity and mortality. Although ECD and associated vascular abnormalities in patients with CKD have been known for decades [2], [3] their causes remain insufficiently understood. It has been proposed that accumulation of homocysteine, overproduction of reactive oxygen species, and/or guanidine compounds and other uremic toxins are the culprits (rev in: [4]). However, the fact that ECD develops at earlier, pre-uremic stages of CKD effectively downplays the contribution of these hypothetical culprits.

Perhaps the most powerful independent contributor to ECD is asymmetric dimethylarginine (ADMA), which is elevated long before the decline in GFR. This guanidino compound, fulfilling many characteristics of “uremic toxin”, is elevated in the course of renal disease and has been found to be the second most valuable (after the patient’s age) predictor of cardiovascular events and mortality in CKD patients, as well as in general population [5], [6]. This endogenous inhibitor of nitric oxide synthases (competing with the substrate L-arginine) arises from the degradation of methylarginine residues in proteins, generating daily >60 mg of ADMA, of which 50 mg are metabolized by dimethylarginine dimethylaminohydrolases (DDAH) and the rest is excreted in the urine [7], [8]. Vascular endothelium is extremely sensitive to ADMA: its infusion elevates blood pressure and peripheral resistance at concentrations equivalent to those seen under pathologic conditions [9].

Inhibition of eNOS and/or deficiency of bioavailable NO are the most common and consistent causes of ECD leading to vasculopathy and defective angiogenesis [10], [11], [12].

L-NMMA treatment, akin to the elevation in ADMA levels, results in NOS uncoupling typified by enhanced generation of superoxide and attenuated NO production [13]. Importantly, increased generation of superoxide anion appears to be the primary cause of the impaired vasorelaxation to acetylcholine in the aorta of mice treated with sub-pressor dose of L-NMMA [14].

Our recent studies employing DIGE of isolated renal microvasculature from L-NMMA-treated animals showed decreased expression of 2 mitochondrial enzymes participating in oxidative phosphorylation: aconitase-2 and enoyl-CoA hydratase-1, potentially leading to truncation of the Krebs cycle and manifesting in the normoxic inhibition of Krebs cycle and induction of glycolysis – hallmarks of Warburg effect seen in tumor cells [15]. As in the case of tumors, this was associated with elevated lactate levels in the plasma.

In the present study, we argued that L-glutamine (GLN), a precursor of α-ketoglutarate, and an FDA-approved nutritional supplement used in critically ill patients, in intensive care units, in patients with cancer and hematologic disorders [16], [17], [18] may have beneficial effect on intermediary metabolism in the model of chronic endothelial dysfunction. GLN is considered an essential amino acid during certain diseases and in several stress situations [19], [20], [21]. L-Glutamine plays a central and quantitatively substantial role in the intermediary metabolism of carbon skeletons and amino groups, and also serves as a precursor for other important pathways. Deamination of glutamine yields NH3 and glutamate which is dehydrogenated or transaminated in α-ketoglutarate to enter in the Krebs cycle. Intestinal absorption of GLN is maintained at 70–80%, and its plasma levels can be monitored. Known effects of supplemental GLN are multiple: improvement of glucose utilization in insulin resistance, stimulation of HSP 70, anti-inflammatory and immunomodulatory actions, enhancement of glutathione synthesis, and stimulation of anabolic processes [22]. GLN is also an essential mitochondrial substrate implicated in protecting cells from reactive oxygen species, preserving α-ketoglutarate dehydrogenase activity and enhancing cell ATP content [23]. GLN is considered a potent anaplerotic molecule capable of replenishing intermediates of the Krebs cycle [24], [25] and its use for therapy of conditions where Krebs cycle activation is desired has been advocated.

Based on this knowledge we hypothesized that L-glutamine supplementation in mice chronically treated with non-pressor doses of L-NMMA, a model of early “asymptomatic” endothelial dysfunction [14], [26], [27] may serve to refuel the distal portion of truncated Krebs cycle, with the possibility of restoring vascular functions.

## Materials and Methods

### Ethics Statements

The animal study protocol was in accordance with the National Institutes of Health Guide for the Care and Use of Laboratory Animals and approved by the New York Medical College’s Animal Care and Use Committee. The Laboratory Animal Complex of the Comparative Medicine at the New York Medical College is registered with the U.S. Department of Agriculture (USDA) and the New York State Department of Health.

### Animal Studies

Male FVB mice, aged 12 weeks, received a non-pressor dose of L-NMMA (0.3 mg/kg/day) in drinking water for 2 months. A randomly selected subgroup of these mice received glutamine supplementation at concentrations 1, 10, and 30 µg/ml. Caveolin-1 (−/−) and caveolin-1 overexpressing transgenic mice were generated by Lisanti’s laboratory and treated similarly with L-NMMA and glutamine supplementation. L-NMMA (N^G^-Monomethyl-L-arginine monoacetate, D-NNMA (N^G^-Monomethyl-D-arginine.monoacetate), and ADMA (N^G^, N^G^-Dimethyl-L-arginine dihydrochloride) were obtained from Alexis Biochemicals (San Diego, CA). There were no differences in the initial body weight among the groups, and the food intake or body weight after chronic GLN or LNMMA feeding did not differ between the groups. At the time of sacrifice, thoracic aortic rings were prepared and the acetylcholine-induced vasorelaxation was examined. Kidneys were removed and microvasculature was isolated using sieving technique exactly as detailed in the previous study [14]. Blood was collected through left ventricular puncture. Renal vascular lysates and plasma were examined using LC-MS/MS spectrometry for all detectable metabolites and multiplex analysis. Serum samples were examined for creatinine concentration (Cayman, Ann Arbor MI.) and cytokines (multiplex biomarker analysis using Linco Inc, St. Louis, MO). Daily urine output was evaluated by sampling urine collected using mouse metabolic cages. Urine albumin and creatinine concentrations were measured using enzyme-linked immunosorbent assay (ELISA) and creatinine kit (Cayman, Ann Arbor MI.) to obtain protein:creatinine ratio.

### Acetylcholine-induced Vasorelaxation of Aortic Rings

The descending thoracic aorta from FVB mice, treated or not treated with L-NMMA and receiving glutamine supplementation, was segmented into cylindrical segments which were mounted on a wire-myograph containing Krebs buffer gassed with 95% O_2_–5% CO_2_ for recording of isometric tension [28]; the vessels were preconstricted with phenylephrine to 70% of maximal response and used for assessment of acetylcholine (0.001–100 µmol/L)-induced vasorelaxation.

### Untargeted Analysis of Metabolites (50–1000 Daltons)

Plasma samples were diluted 1∶20 in 70% acetonitrile/dH2O containing 0.2% acetic acid. The diluted samples were briefly vortexed and centrifuged to pellet precipitated proteins. Sample supernatants were transferred to autosampler vials for analysis by HPLC-MS and HPLC MS/MS. The LC/MS setup was the same as described previously [29].

### Metabolomics Data Processing and Analysis

Raw data files were processed by Agilent MassHunter Qualitative Analysis Software and analyzed by statistical analysis in Mass Profiler Professional as described [29]. (Agilent Technology, MPP, version B2.02). Briefly, MassHunter Qualitative2 Analysis untargeted molecular feature extraction (MFE) generates features (compounds/metabolites) based on the elution profile of identical mass and retention times, within a defined mass accuracy (5 ppm). Aligned molecular features detected in all biological replicates of at least one group were directly applied for statistical analysis across treatment groups by MPP. The Benjamini Hochberg FDR correction was applied for multiple testing correction of p-values in one-way ANOVA (corrected P<0.05). Uncorrected P-value was used when individual metabolites were manually inspected for statistical significance between two groups(e.g simple student t-test.).

Differential metabolites were initially searched against an in-lab annotated METLIN Personal Metabolite Database (Agilent Technologies), based on accurate monoisotopic neutral masses (<5 ppm). A molecular formula generator (MFG) algorithm in MPP was used to generate and score empirical molecular formulae based on a weighted consideration of monoisotopic mass accuracy, isotope abundance ratios, and spacing between isotope peaks. Notably, MFG imposes additional constraints on the list of candidate molecular formulas detected by a METLIN database search. A putative compound ID was tentatively assigned when METLIN and MFGconcurredfor a given candidate. Tentatively assigned compounds were verified based on a match of LC retention time and/or MS/MS fragmentation patterns to pure molecular standards. As no repository exists for metabolomic data, the authors will make the results of this study available to all interested researchers.

### Circulating Levels of Cyto- and Chemokines

The multiplex mouse cardiovascular diseases biomarkers panel and the Cytokines/Chemokines panel were used (MCVD1-67AK 4 plex, MCyt/Chem 13Plex, Linco Inc, St. Louis, MO) for the simultaneous quantification of the following analytes: soluble E-Selectin (sE-Selectin), soluble ICAM (sICAM-1), soluble VCAM-1 (sVCAM), Matrix Metalloproteinase-9 (MMP-9) and 13 Soluble Cytokines (MIP-1α, GMCFS, MCP1, KC, RANTES, IFNγ, IL1β, IL1α, GCSF, IP10, IL-10, TNF-α). All examined analytes had been tested individually and in combination to ensure that there were no cross-reactions. Briefly, the multibiomarkers and cytokine standards were resuspended in the assay buffer and then differentially serially diluted. Twenty-five µl of standard, Quality Controls or sample were added to each well of a 96-well plate with 25 µl of the bead solution. Each plate was sealed, covered with aluminum foil and incubated overnight (16–18 h) with agitation on a shaker platform at 4°C. The plates were washed twice with 200 µl/well of washing buffer, buffer being removed by vacuum filtration between each wash. This was followed by addition of 25 µl of a detection antibody cocktail into each well and incubation at room temperature for 1.5 h. Streptavidin-phycoerythrin solution (25 µl) was added to each well and incubated at room temperature for 30 min. The multianalyte composition was then analyzed using a LuminexIS100 analyzer (Luminex Inc, Austin TX). The data were evaluated as Median Fluorescence Intensity (MFI) using appropriate curve-fitting software (Luminex 100IS software version 2.3). A 5-parameter logistic method with weighing was used.

### Statistical Analysis

All other quantitative data were determined as the mean value ± SEM. Statistical analyses of data were performed by ANOVA for multiple-group means or by Student’s *t* test for comparisons between two group means. Statistical significance was set at the level of *P*<0.05.

## Results

**Figure 1:**
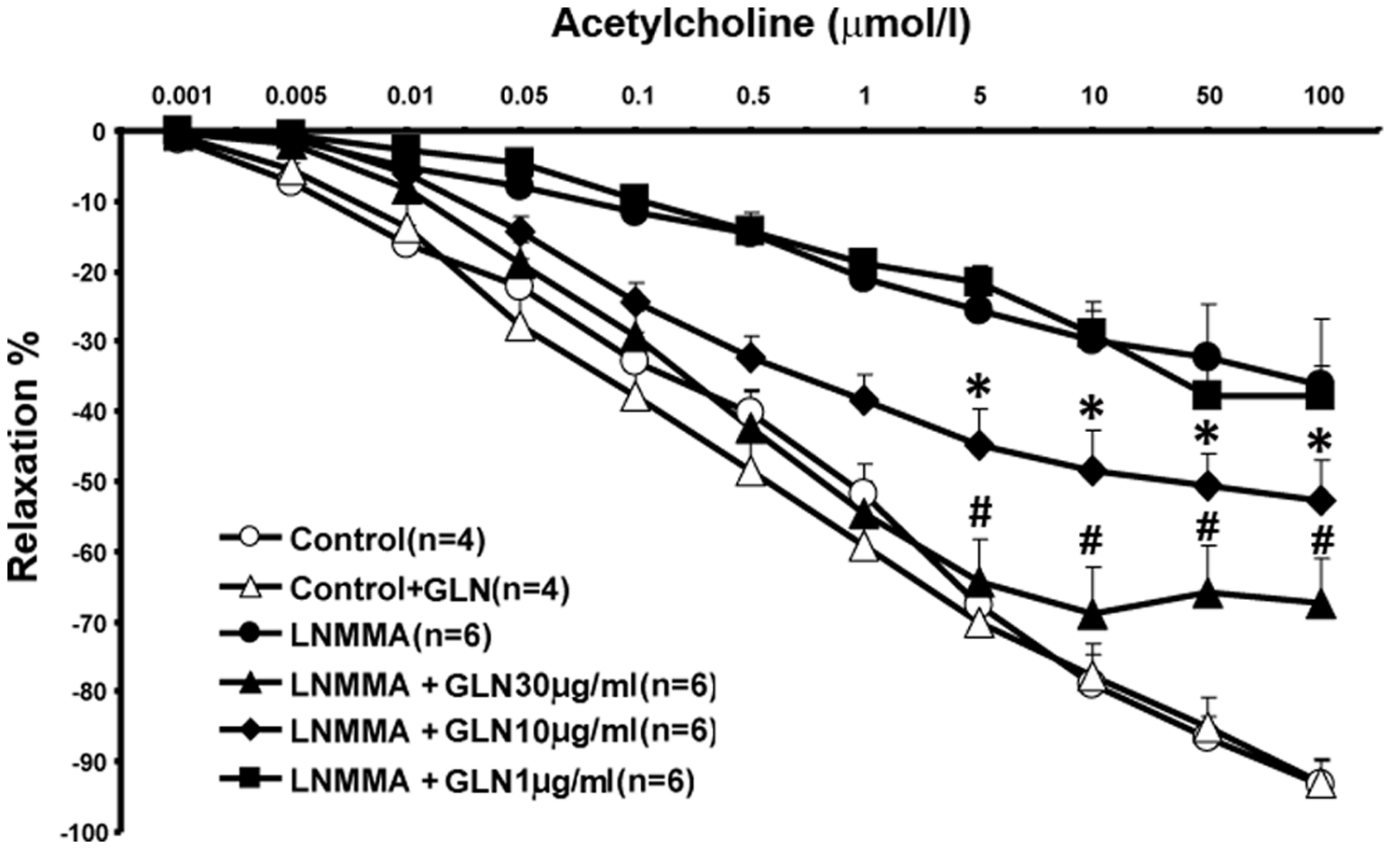
Chronic administration of glutamine dose-dependently improves the acetylcholine-induced endothelium-dependent vasorelaxation in L-NMMA-treated mice. Both L-NMMA and glutamine, diluted in drinking water, were administered for 2 months.

**Figure 2:**
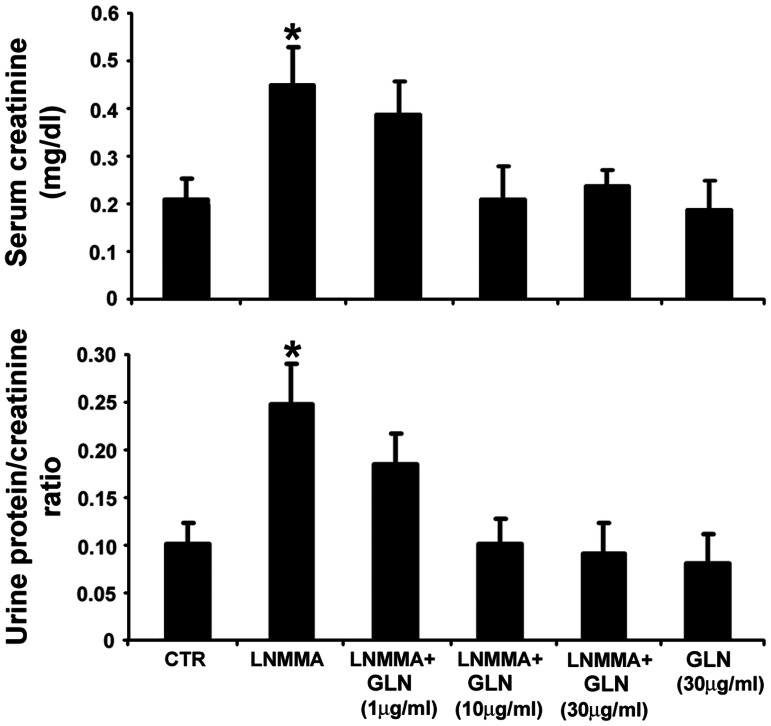
Renal function in mice receiving L-NMMA and treated with L-glutamine. The graphs summarize results of serum creatinine and albumin:creatinine ratio in the urine of control mice and mice receiving a non-pressor dose of L-NMMA with and without 1, 10, and 30 µg/ml L-glutamine. Note that glutamine alone had no effect on either of these parameters of renal function under control conditions. Mice receiving L-NMMA had a mild elevation in serum creatinine and proteinuria. Those animals which were simultaneously treated with L-glutamine at 10 and 30 µg/ml (but not 1 µg/ml) showed normalization of both parameters of renal function.

Previous studies [14], [27] demonstrated that chronic administration of non-pressor doses of L-NMMA (0.3 mg/ml of drinking water daily for 2-months) results in a mild endothelial dysfunction. As shown in **Figure 1**, this finding was reconfirmed using an ex vivo aortic ring vasorelaxation assay. Moreover, concomitant treatment with glutamine at doses 1, 10 and 30 µg/ml of drinking water, significantly protected against the otherwise impaired acetylcholine-induced relaxation of aortic rings. This protective effect of GLN was dose-dependent. In addition, mice receiving L-NMMA exhibited signs of nephropathy: a mild significant elevation in serum creatinine and proteinuria (**Figure 2**). These findings suggest the existence of a link between the impaired metabolic state of the vascular endothelium and suppressed nitric oxide-dependent vasorelaxation associated with the developing nephropathy.

**Figure 3:**
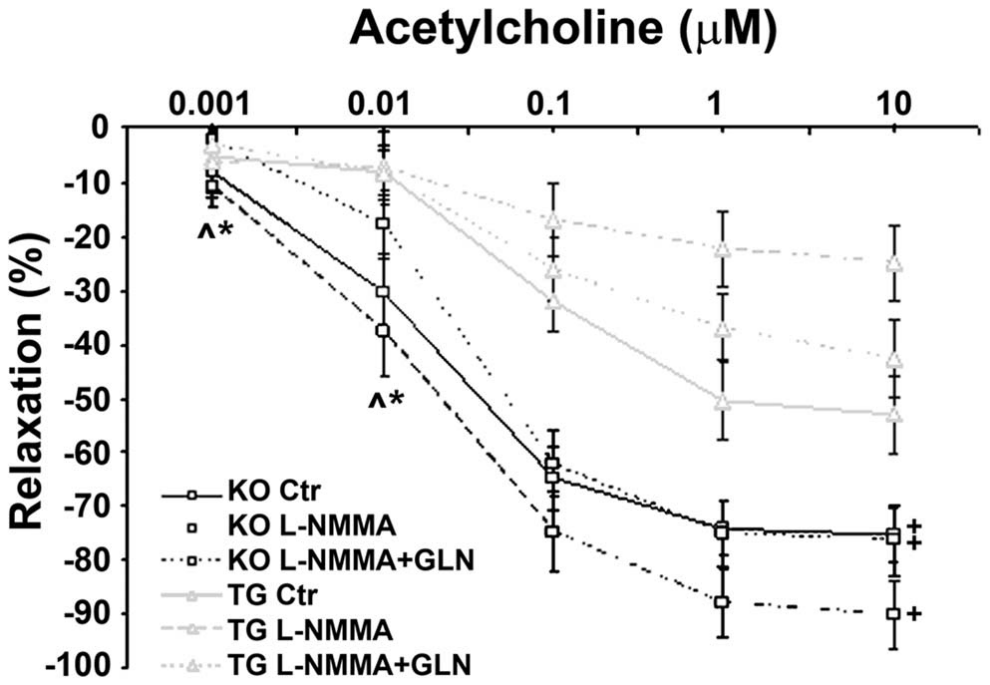
Glutamine supplementation dose-dependently improves acetylcholine-induced vasodilatatory response of thoracic aortic rings in caveolin-1 transgenic but not in knock-out mice. +p<0.05; KO vs. TG (same treatment; only Ach 0.1 µM, 1 µM, 10 µM). ∧p<0.05; KO (Ctrl, L-NMMA, L-NMMA+Glutamine) vs. Ach 0.1 µM, 1 µM, 10 µM *p<0.05; TG (Ctr) vs. Ach 0.1 µM, 1 µM, 10 µM n = 6.

To further examine this link, we performed similar studies in caveolin-1(−/−) and caveolin-1 overexpressing transgenic mice. It is well-established that the function of eNOS is dependent on caveolin-1 [30]. There is evidence that both manipulations of caveolin-1 expression result in perturbations of vascular functions [31] [32], [33]. Under basal conditions, aortic rings from caveolin-1 knockout mice, previously shown to overproduce nitric oxide [34] exhibited a robust vasorelaxation in response to acetylcholine and this response was not affected by non-pressor doses of L-NMMA with or without GLN supplementation (**Figure 3**). In contrast, aortic rings obtained from caveolin-1 transgenic mice exhibited a significant suppression of vasorelaxing effect of acetylcholine, which became even more profound in mice treated with non-pressor doses of L-NMMA (**Figure 3**). When these mice were provided with GLN supplementation, vasorelaxing action of acetylcholine was partially rescued. These findings provided additional evidence to support the existence of a link between the metabolic status of vascular endothelium and nitric oxide-dependent vasorelaxation.

**Figure 4:**
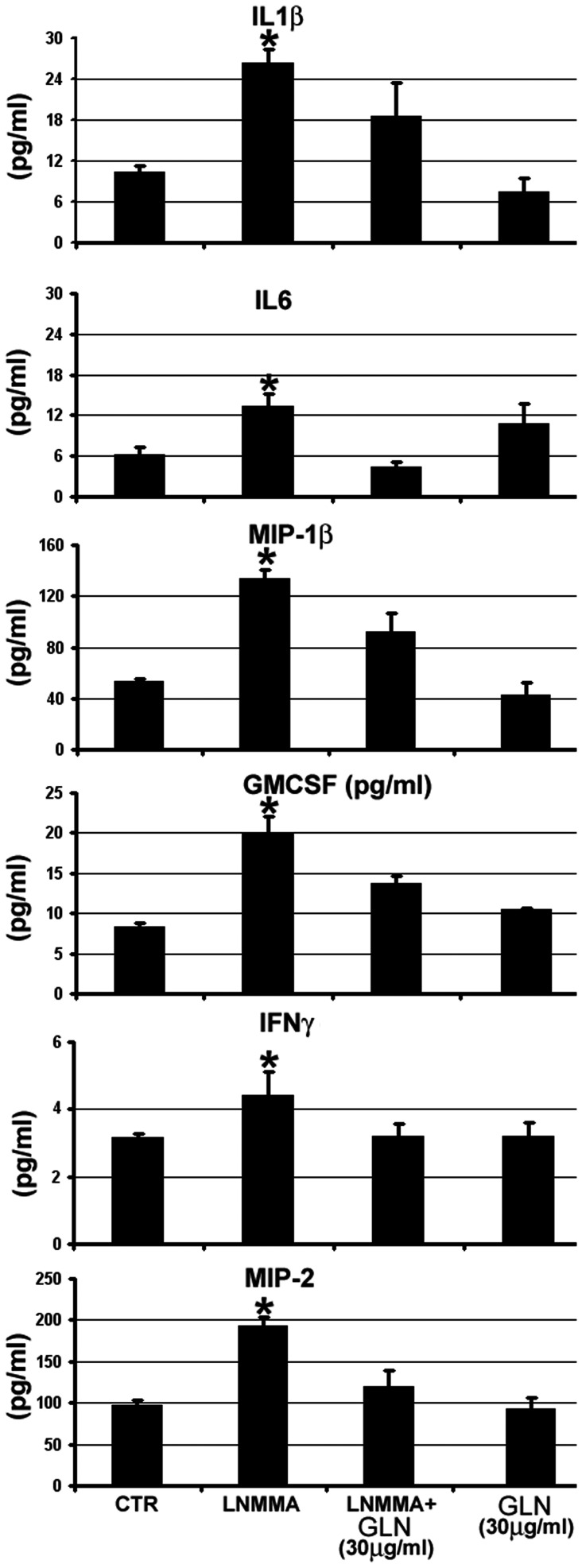
Results of cyto−/chemokine screening of plasma in mice receiving L-NMMA and treated with 30 µg/ml L-glutamine. Experiments were performed using a Luminex platform allowing detection of 21 cyto−/chemokines listed in the Methods. Only those components of the screen that showed statistically significant differences between the groups are depicted.

In view of the previous demonstration of immunomodulatory effect of GLN, we next sought to examine the possibility of immunologic effects of GLN supplementation in mice chronically treated with L-NMMA. Toward this end, multiplex analysis of cyto- and chemokines was carried out. Levels of interleukins IL1β, IL-6, as well as MIP-1β, MIP-2, INF-γ, and GM-CSF all showed significant increase in L-NMMA-treated mice (**Figure 4**). GLN supplementation on its own did not alter levels of cytokines in control mice. In contrast, addition of GLN to L-NMMA-treated mice resulted in a significantly decreased the level of IL-6 and GM-CSF and blunted (p = 0.1–0.15) L-NMMA-induced elevation of MIP-1β, MIP-2, INF-γ, and soluble E-selectin. These findings argue in favor of anti-inflammatory effect of GLN supplementation in L-NMMA-treated mice.

**Figure 5:**
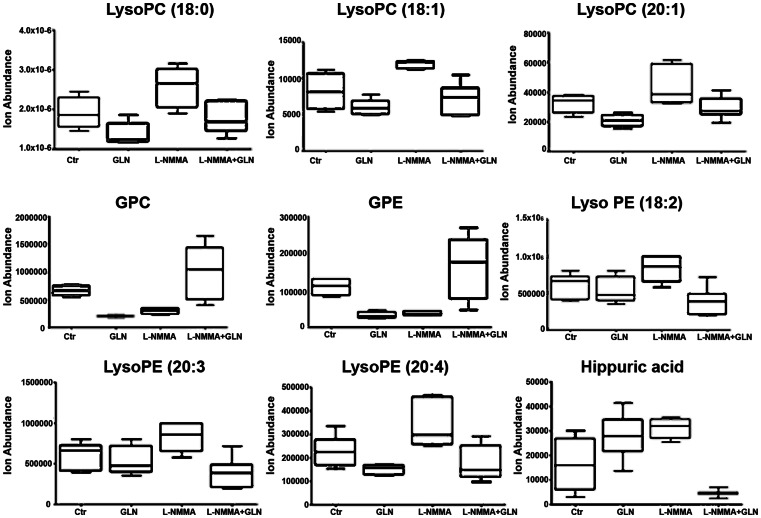
LNMA-induced plasma lysophospholipid changes that were offset by glutamine. Bioactive lysophosphocholines are involved in key pathobiological processes associated with vascular disease. Several lipid metabolites (glycerophosphocholine, glyceroethanolamine, lysophosphatidylethanolamines [22∶6 and 18∶2]) and hippuric acid exhibited the required dynamics.

In an attempt to uncover metabolites potentially responsible for a) L-NMMA-induced ECD and b) GLN rescue of ECD, as judged by the acetylcholine-induced vasorelaxation, we next performed metabolomic profiling of plasma and isolated microvascular samples obtained from control and L-NMMA-treated mice with or without GLN supplementation. The number of metabolites of molecular size 50–1000 Da detectable in the plasma of these mice was as follows: 3453 molecules could be detected with 100% frequency in mice from at least one treatment group (**Figure S1**). Among these molecules, 40 were found to be differentially expressed in a 4-way comparison of Control vs. L-NMMA vs. GLN vs. GLN+L-NMMA groups (one-way ANOVA, Benjamini-Hochberg FDR corrected P<0.05). Principal component analysis PCA clearly differentiated all treat groups (**Figure** **S1B**) and unsupervised hierarchical cluster analysis revealed a clear and reproducible pattern of within-group metabolite expression similarities and between-group differences (**Figure S1C**). L-NMMA+GLN and control shared 41 differential metabolites when compared L-NMMA (**Figure S1D**, uncorrected P<0.05, fold change >1.5). To narrow the field of potential candidates for the observed effects of L-NMMA and glutamine, attention was focused on those, which showed a reciprocal dynamics between a) control and L-NMMA and b) L-NMMA and L-NMMA with GLN. Such a strategy has a higher probability of uncovering metabolite(s) of interest. A group of metabolites fitting these imposed requirements is presented in **Figure 5**. Notably, several lipid metabolites (glycerophosphocholine, glyceroethanolamine, lysophosphatidylethanolamines [22∶6 and 18∶2]) and hippuric acid exhibited the required dynamics.

**Figure 6:**
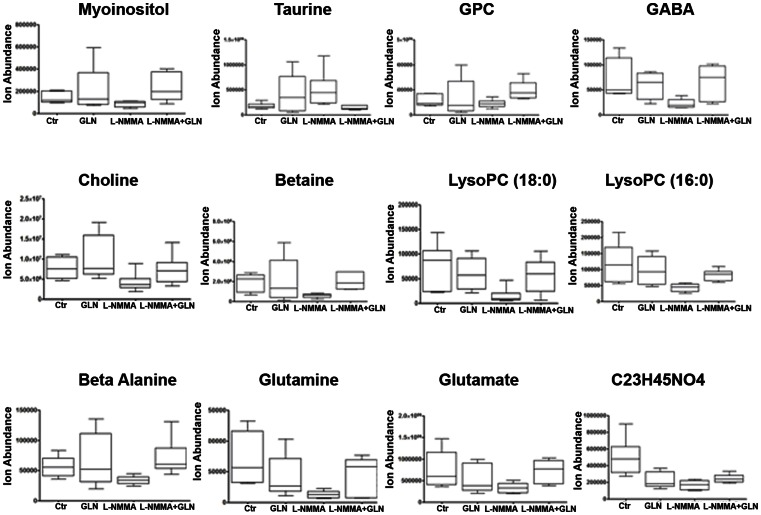
LNMA-induced kidney blood vessel metabolite changes that were offset by glutamine. L-NMMA-induced renal microvascular metabolite changes that were offset by GLN supplementation included myoinositol, GPC, betaine, and taurine wich was the only osmolyte up-regulated in L-NMMA-treated mice (as compared to down-regulation of GPC, betaine and myoinisitol) and compensated by GLN treatment. GABA and alanine, potential inhibitors of taurine transport, were also down-regulated in L-NMMA treated mice.

Metabolic profiling of renal microvasculature detected 1400 aligned features in all 6 replicates of at least one treatment group by untargeted molecular feature extraction (**Figure S2A**). Principal component score plot analysis showed an L-NMMA-dependent clustering and separation of control and treatment groups (**Figure S2B**). Unsupervised hierarchical clustering showed clustering and branching of mouse kidney vessel metabolites by GLN treatment (**Figure S2C**). As summarized by the Venn diagram, 37 differential metabolites in L-NMMA+GLN vs L-NMMA and Control vs L-NMMA (uncorrected P<0.05, fold change>2) were shared. L-NMMA-induced renal microvascular metabolite changes that were offset by GLN supplementation (**Figure 6**) included myoinositol, GPC, betaine, and taurine (four different renal organic osmolytes which provide the safety factor of redundancy of their protective effect on kidney function). Notably, taurine was the only osmolyte up-regulated in L-NMMA-treated mice (as compared to down-regulation of GPC, betaine and myoinisitol) and compensated by GLN treatment. GABA and alanine, potential inhibitors of taurine transport, were also down-regulated in L-NMMA treated mice.

**Figure 7:**
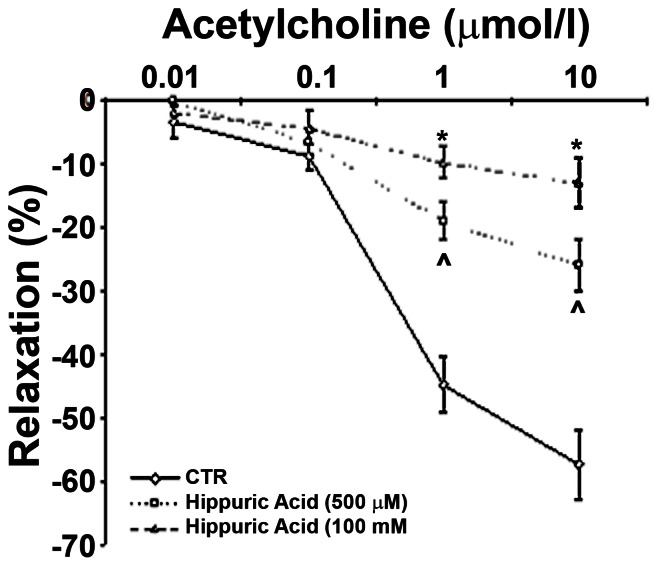
Hippuric acid dose-dependently suppresses acetylcholine-induced vasorelaxation of aortic rings. Thoracic aortic rings were incubated in the presence of hippuric acid during equilibration period and then subjected to escalating concentrations of acetylcholine. *p<0.05 vs. Control & Hippuric Acid (500 µM) ∧p<0.05 vs. Control, n = 4.

Hippuric acid is a well-known “uremic toxin” [35], thus its elevation in non-uremic mice, albeit with endothelial cell dysfunction, was unexpected. To ascertain potential effects of hippuric acid on vasomotion, in the next series of experiments we employed thoracic aortic rings to examine its vasoconstrictor effects and its influences on vasorelaxation. Addition of hippuric acid at concentrations of 100 um-1 mM to the incubation bath did not produce vasoconstriction (not shown). In contrast, hippuric acid significantly and dose-dependently attenuated acetylcholine-induced relaxation of aortic rings (**Figure 7**). These findings establish the hitherto unknown action of hippuric acid, a putative “uremic toxin”, to alter vasorelaxation of large vessels. These observations provide a potential link between the accumulation of hippuric acid, even in pre-uremic animals, and the early development of vasculopathy.

## Discussion

Data presented herein establish that in mice with isolated endothelial cell dysfunction induced by chronic administration of non-pressor doses of L-NMMA GLN supplementation ameliorates impaired vasorelaxation and proteinuria, blunts elevation in the level of “uremic toxin” hippuric acid, and mitigates elevation in pro-inflammatory cytokines.

Though it was not the purpose of this study to examine molecular mechanisms of GLN action, the previously described contribution of GLN to glutathione synthesis and its maintenance in reduced form by providing a source of reducing equivalents like NADPH [36] is highly relevant to the present dataset. A similar end-effect can be observed in erythrocytes, where GLN plays a role of antioxidant and preserves NADPH level required for glutathione recycling [37]. Consistent with our previous demonstration of enhanced oxidative stress in the L-NMMA model of endothelial cell dysfunction [14], the above-mentioned antioxidant defense by GLN supplementation can explain some of the observed benefits of this therapy: prevention of eNOS uncoupling and generation of peroxynitrite. An additional potential mechanism of beneficial vascular effect of GLN supplementation can be the consequence of restored Krebs cycling of intermediates with the activation of malate shuttle and enhanced production of NADPH, a co-factor of endothelial nitric oxide synthase. Furthermore, the ability of GLN to be converted to L-citrulline [38], with the latter converted to L-arginine, may underlie the observed restoration of endothelium-dependent vasorelaxation. Such a necessity may occur in situations accompanied by uncoupling of eNOS, as it occurs in cultured porcine aortic endothelial cells where L-NMMA stimulated L-arginine efflux via y+ transporter, potentially depleting its intracellular pool [39] and further exacerbating eNOS uncoupling.

The whole subject of GLN effects on eNOS is strewn with controversies. Early studies demonstrated that GLN inhibits release of endothelium-derived relaxing factor [40], but did not interfere with the uptake of L-arginine, rather inhibited its generation [41]. In cultured endothelial cells and in aortic rings, L-glutamine inhibited bradykinin-induced NO production, but increased NO production in response to a calcium ionophore; in either case effect was not due to modulation of eNOS activity [42]. In rat aortic rings, however, L-glutamine increased NO formation and vessel relaxation in eNOS-dependent manner [43]. In rabbits with thromboxane-A_2_ mimetic-induced pulmonary hypertension, GLN administration increased pulmonary artery pressure, but the opposite effect was documented in animals pretreated with L-NMMA [44].

In rats on cardio-pulmonary bypass, GLN administration reduced plasma levels of IL-6 and IL-8, improved myocardial respiration, increased the levels of HSP-70, preserved the activity of eNOS and attenuated induction of iNOS [45]. Notwithstanding these uncertainties generated in cultured cells or in acute experiments, in our case of chronic inhibition of NO production, GLN supplementation alleviated vasculopathy, reduced pro-inflammatory cytokines, and improved microcirculatory functions, as judged from the reduced proteinuria and serum creatinine.

One of the unexpected findings of untargeted metabolomic analysis is related to the L-NMMA-induced reduction of vascular GLN. In this vein, peroxynitrite, a constantly elevated by-product of endothelial dysfunction, decreases the activity and expression of glutamine synthetase due to nitration of tyrosine residues in the active site [46]. Moreover, GLN consumption may be elevated in mice with chronic L-NMMA treatment to compensate for the reduced reduced cellular L-arginine levels [39], and the developing deficiency is prevented by GLN supplementation, thus preserving its level and above-mentioned functions.

In patients with chronic kidney disease, the level of hippuric acid increases from 2.2 µM in control to 160 µM in far-advanced disease [47]. Hippurate has been shown to inhibit glucose utilization in striated muscles [48] thus contributing to muscle weakness and to accelerate the progression of chronic kidney diseases [49]. Since the renal excretion of hippurate represents the major pathway for its elimination [50], the finding of its elevation in our non-uremic mice displaying only mild renal impairment was rather unexpected. Similarly unexpected, but consistent with our findings, was the mass-spectrometric detection of elevated levels of hippuric acid released by the human vascular wall subjected to inflammation and oxidative stress as occurs in athrothrombotic aneurismal areas [51]. These and our findings argue in favor of the increased metabolic rate of hippurate synthesis during syndromes associated with endothelial cell dysfunction.

Notably, L-NMMA treatment failed to alter plasma levels of Krebs cycle intermediates, suggesting that the ECD-associated Krebs cycle suppression in renal vasculature may be relatively restricted to the vascular endothelium and insufficient to globally influence metabolite levels in the circulation. This is not entirely surprising. Although it is currently impossible to detect the actual size and any potential improvement in the α-ketoglutarate and other intermediates’ pool (according to Krebs, “the fate of the label does not allow predictions to be made about the net fate of the labeled metabolites”), relative levels can be assessed based on the functional improvement [52]. This functional improvement was precisely what we have demonstrated here: GLN supplementation reduced manifestations of vasculopathy and pro-inflammatory profile associated with endothelial cells dysfunction.

**Figure 8:**
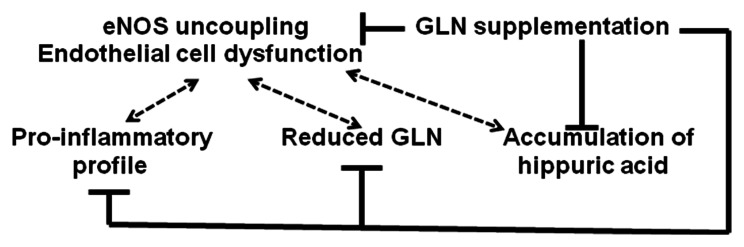
Cartoon summarizing major functional and metabolomic findings and proposed pathways of L-glutamine supplementation-induced amelioration of vascolopathy (see text for the details).

Note: After this paper was accepted for the publication, 2 relevant papers have been published. Jeong and colleagues [53] demonstrated that DNA damage response is associated with decreased glutamine/glutamate conversion into alpha-ketoglutarate via sirtuin-4-dependent inhibition of glutamate dehydrogenase. This response is required for the DNA-damaged cell to enter cell cycle arrest. Reid et al. [54] demonstrated that glutamine deprivation results in reduction of reduced glutathione, increased oxidative stress and activation of transcription factor p53. Both described pathways of glutamine deficiency-induced cell cycle arrest and p53 activation may be relevant to the proposed mechanisms of action of glutamine supplemetation in dysfunctional endothelial cells.

In conclusion, GLN supplementation in mice with endothelial cell dysfunction improves vasculopathy, nephropathy and a profile of circulating pro-inflammatory mediators. Curiously, endothelial dysfunction *per se* causes GLN deficiency, thus explaining in part the beneficial effect of its supplementation. Based on these findings and previous investigations by others, we propose an up-dated mechanistic explanation of the observed effects of GLN supplementation on microvascular function in L-NMMA-treated mice (**Figure 8**). The pathways potentially involved in the observed end-effect of GLN supplementation of L-NMMA-treated mice with endothelial dysfunction include the cytosolic (replenishing GLN level), systemic (anti-inflammatory, reduction of hippuric acid) and possibly mitochondrial (Krebs cycle) components. Based on these findings, we are currently initiating pilot clinical studies of glutamine supplementation in patients with chronic kidney diseases. In addition, data show that plasma metabolite profiling offers the potential to provide a molecular definition of ECD, diagnose ECD subtypes, assess severity and monitor the efficacy of new therapies. Furthermore, it has a potential to disclose hitherto unknown mediators of progression of disease, as is the case with the discovery of 1) GLN deficiency in mice with endothelial dysfunction and 2) hippuric acid elevation long before the uremic stage of kidney disease, both contributing to developing vasculopathy and organ failure.

## Supporting Information

Figure S1 Metabolite profiling identifies differentially expressed metabolites among untreated mouse plasma and treatment groups of LNMA, glutamine, LNMMA+glutamine. (A) 3453 aligned features were detected in all 6 replicates of at least one treatment group by untargeted molecular feature extraction. 40/3453 were found to be differentially expressed in a 4-way comparison of Control vs. L-NMMA vs. GLN vs. GLN+L-NMMA groups (one-way ANOVA, Benjamini-Hochberg FDR corrected p<0.05) (B) PCA score plot shows a drug-dependent clustering and separation of treatment groups and control using the 40 metabolites from one-way ANOVA. (C) Unsupervised hierarchical clustering shows clustering and branching of mouse plasma by glutamine treatment. (D)Venn diagram shows 41 shared differential metabolites in LNMA+Glutamine vs LNMA and Control vs LNMA (uncorrected P<0.05, fold change >1.5).
(TIF)

Figure S2 Metabolite profiling identifies differentially expressed metabolites among mouse kidney vessels of untreated group and treatment groups of LNMA, glutamine, LNMA+glutamine. (A) 1400 aligned features were detected in all 6 replicates of at least one treatment group by untargeted molecular feature extraction. (B) PCA score plot shows a drug-dependent clustering and separation of treatment groups and control. (C) Unsupervised hierarchical clustering shows clustering and branching of mouse kidney vessel metabolites by glutamine treatment. (D) Venn diagram shows 37 shared differential metabolites in LNMA+Glutamine vs LNMA and Control vs LNMA (uncorrected P<0.05, fold change >2.0).
(TIF)
