# Supplementary figures and images for: Glutamine Supplementation Alleviates Vasculopathy and Corrects Metabolic Profile in an In Vivo Model of Endothelial Cell Dysfunction
Source: PLoS One. 2013 Jun 11;8(6):e65458. doi: 10.1371/journal.pone.0065458 (PMC3679132; doi:10.1371/journal.pone.0065458)

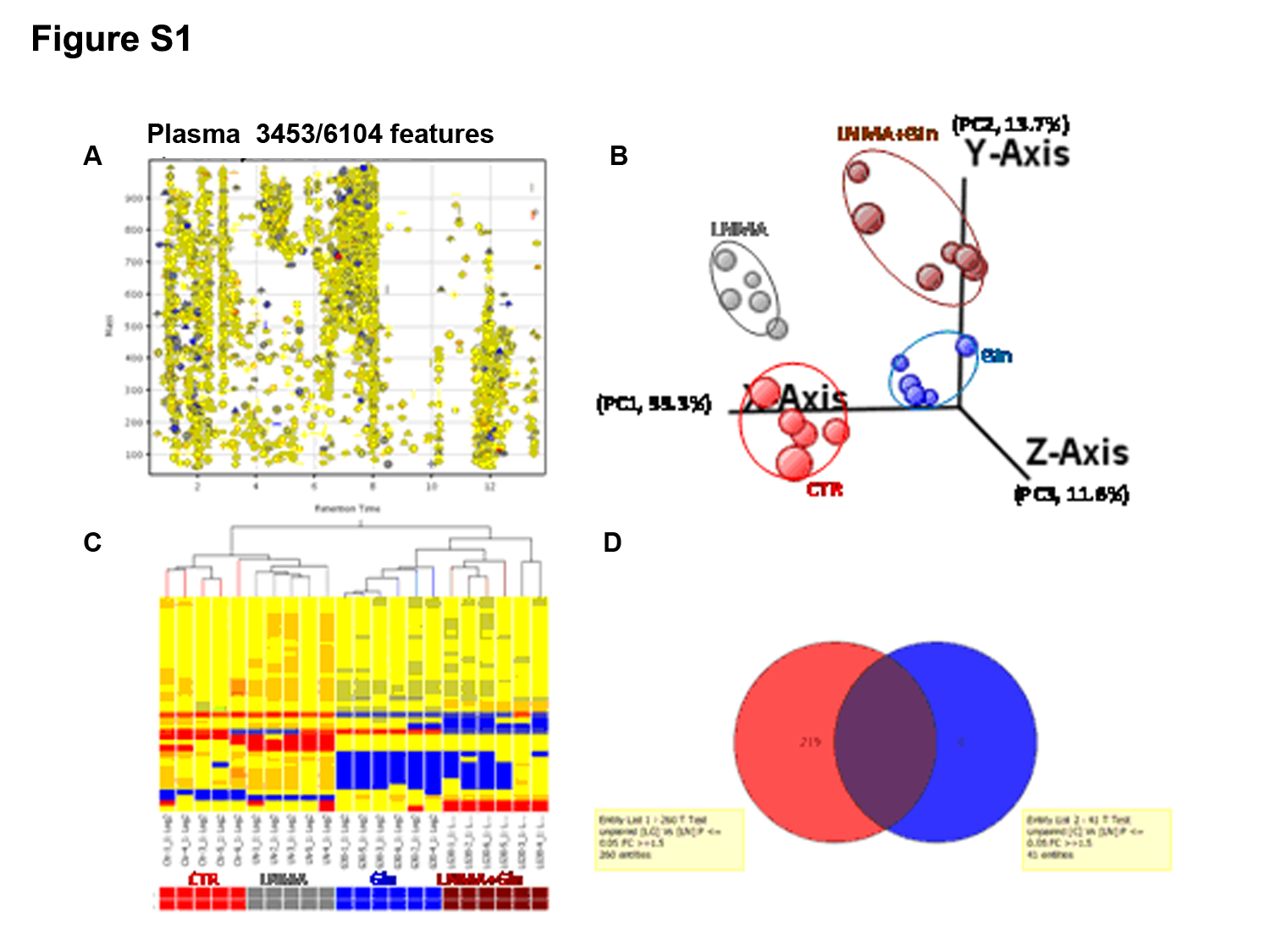

Supplement: Figure S1 — Metabolite profiling identifies differentially expressed metabolites among untreated mouse plasma and treatment groups of LNMA, glutamine, LNMMA+glutamine. (A) 3453 aligned features were detected in all 6 replicates of at least one treatment group by untargeted molecular feature extraction. 40/3453 were found to be differentially expressed in a 4-way comparison of Control vs. L-NMMA vs. GLN vs. GLN+L-NMMA groups (one-way ANOVA, Benjamini-Hochberg FDR corrected p<0.05) (B) PCA score plot shows a drug-dependent clustering and separation of treatment groups and control using the 40 metabolites from one-way ANOVA. (C) Unsupervised hierarchical clustering shows clustering and branching of mouse plasma by glutamine treatment. (D)Venn diagram shows 41 shared differential metabolites in LNMA+Glutamine vs LNMA and Control vs LNMA (uncorrected P<0.05, fold change >1.5). (TIF) [file pone.0065458.s001.tif]

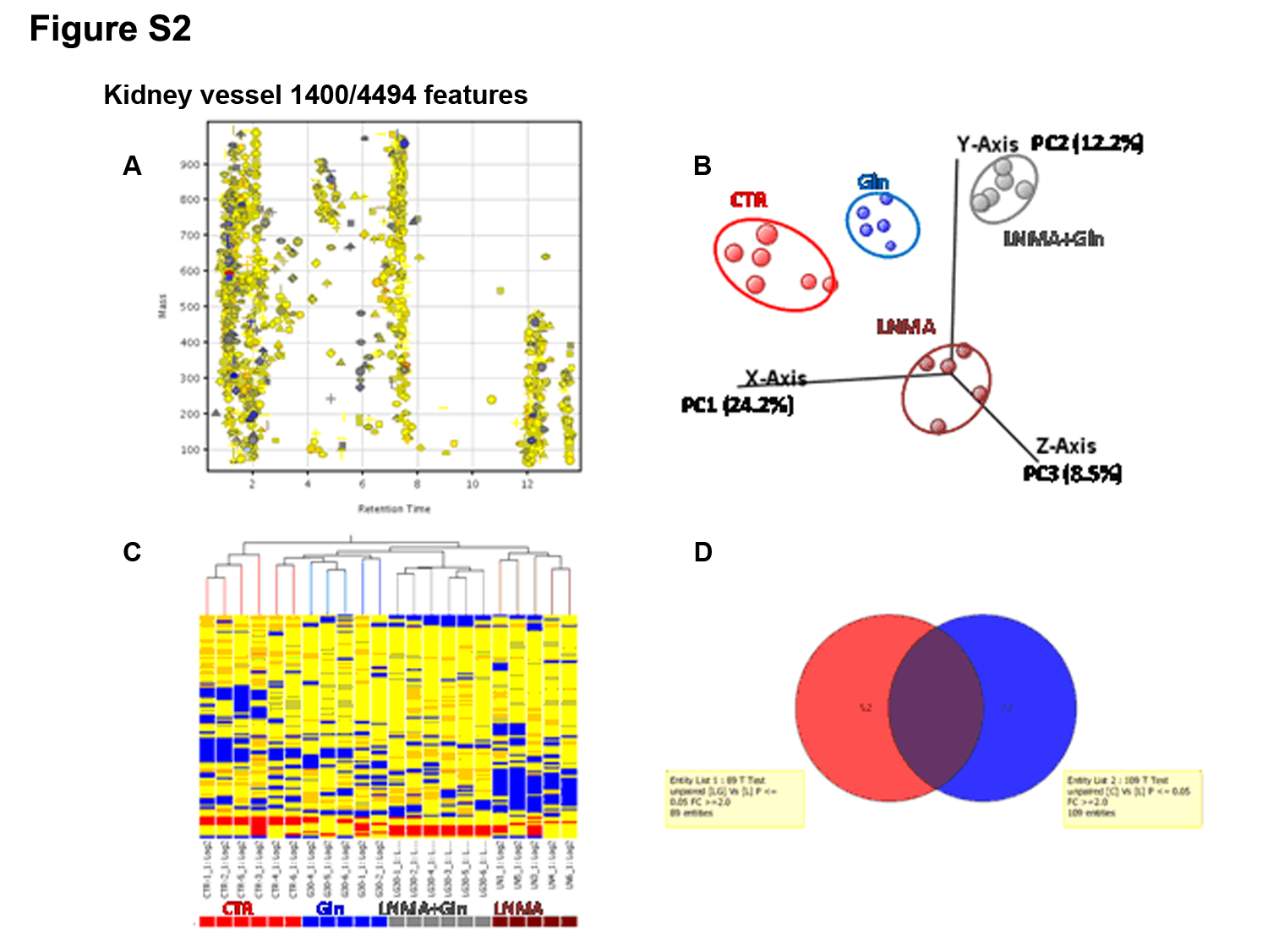

Supplement: Figure S2 — Metabolite profiling identifies differentially expressed metabolites among mouse kidney vessels of untreated group and treatment groups of LNMA, glutamine, LNMA+glutamine. (A) 1400 aligned features were detected in all 6 replicates of at least one treatment group by untargeted molecular feature extraction. (B) PCA score plot shows a drug-dependent clustering and separation of treatment groups and control. (C) Unsupervised hierarchical clustering shows clustering and branching of mouse kidney vessel metabolites by glutamine treatment. (D) Venn diagram shows 37 shared differential metabolites in LNMA+Glutamine vs LNMA and Control vs LNMA (uncorrected P<0.05, fold change >2.0). (TIF) [file pone.0065458.s002.tif]
